# Supplementary material for: Detecting subtle transcriptomic perturbations induced by lncRNAs knock-down in single-cell CRISPRi screening using a new sparse supervised autoencoder neural network
Source: Front Bioinform. 2024 Mar 4;4:1340339. doi: 10.3389/fbinf.2024.1340339 (PMC10945021; doi:10.3389/fbinf.2024.1340339)
Supplement: Supplementary file 1 [file DataSheet1.PDF]

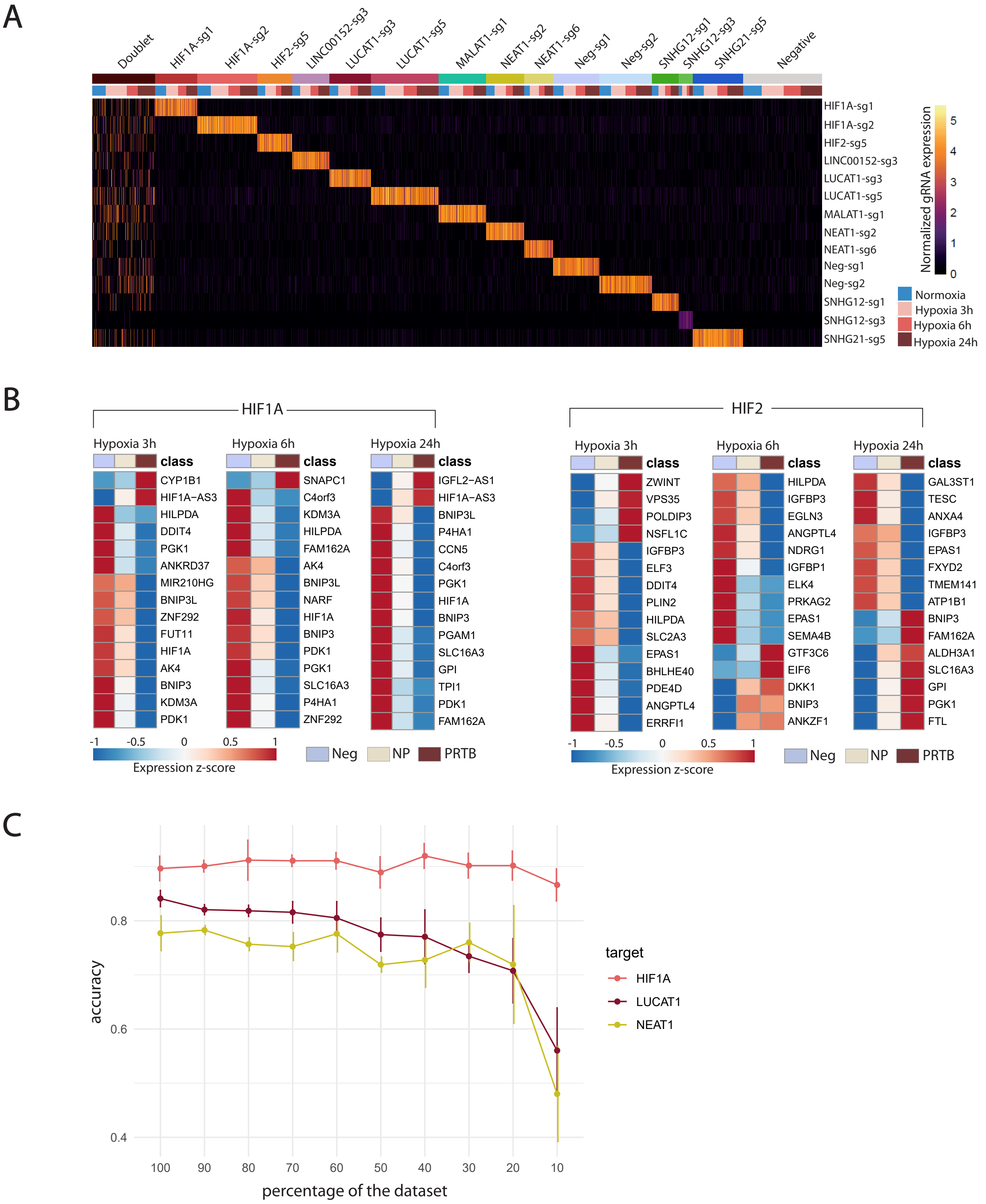

Supplemental Figure : A: Heatmap of gRNA counts in each cell, labelled according to assigned gRNA and condition after demultiplexing. **B:** Average expression of the top 15 genes of the perturbation signature induced by HIF1A (left) and HIF2 (right) inhibition between control (Neg), non-perturbed (NP) and perturbed (PRTB) cells in the 3 time points of hypoxia exposure. **C:** Evolution of classification accuracy by progressively sub-sampling the HIF1A, LUCAT1 and NEAT1 datasets (cells targeted by the respectives gRNA + control cells) in Hypoxia 24h.
